# Contrasting impacts of dry versus humid heat on US corn and soybean yields

**DOI:** 10.1038/s41598-023-27931-7

**Published:** 2023-01-13

**Authors:** Mingfang Ting, Corey Lesk, Chunyu Liu, Cuihua Li, Radley M. Horton, Ethan D. Coffel, Cassandra D. W. Rogers, Deepti Singh

**Affiliations:** 1 Lamont-Doherty Earth Observatory, Columbia University, 61 Rt. 9W, Palisades, NY 10964 USA; 2 Neukom Institute for Computational Science, Dartmouth College, Hanover, NH USA; 3 Department of Geography, Dartmouth College, Hanover, NH USA; 4 School of Oceanography, Shanghai Jiao Tong University, Shanghai, China; 5 Department of Geography and the Environment, Syracuse University, Syracuse, NY USA; 6 School of the Environment, Washington State University, Vancouver, WA USA

**Keywords:** Climate sciences, Climate change, Climate-change impacts

## Abstract

The impact of extreme heat on crop yields is an increasingly pressing issue given anthropogenic climate warming. However, some of the physical mechanisms involved in these impacts remain unclear, impeding adaptation-relevant insight and reliable projections of future climate impacts on crops. Here, using a multiple regression model based on observational data, we show that while extreme dry heat steeply reduced U.S. corn and soy yields, humid heat extremes had insignificant impacts and even boosted yields in some areas, despite having comparably high dry-bulb temperatures as their dry heat counterparts. This result suggests that conflating dry and humid heat extremes may lead to underestimated crop yield sensitivities to extreme dry heat. Rainfall tends to precede humid but not dry heat extremes, suggesting that multivariate weather sequences play a role in these crop responses. Our results provide evidence that extreme heat in recent years primarily affected yields by inducing moisture stress, and that the conflation of humid and dry heat extremes may lead to inaccuracy in projecting crop yield responses to warming and changing humidity.

## Introduction

One of the known consequences of anthropogenic climate change is an increase in both regional and global mean temperatures, as well as significant increases in extreme heat event magnitudes^1^. Recent research has highlighted that combinations of extreme heat and humidity are increasing in frequency with climate change^2,3^ and will continue to increase in the future^4^, posing particular stresses to people and other mammals^5^. Recent studies^6,7^ indicate that for the tropics and midlatitudes, only half of the heat accumulation in the atmosphere since the mid-20th Century is due to sensible heat (i.e., rising air temperature), with the other half coming from latent heat (increased humidity). At the same time, heatwaves are projected to become drier (net loss of surface moisture in terms of precipitation minus evaporation) with warming in some regions^8,9^. Climate change therefore has the potential to alter the moist thermodynamic characteristics of heatwaves as circulation, precipitation, and land–atmosphere interactions respond to rising CO_2_.

Crops are known to experience yield loss when exposed to extremely high temperatures^10,11^. While extreme heat can simultaneously cause direct thermal stress to crops^12,13^, it can also induce indirect moisture stress by raising atmospheric aridity (i.e., vapor pressure deficit)^14,15^. During high-temperature and high-humidity extremes, on the other hand, this indirect moisture stress is somewhat limited. Precipitation and soil moisture further complicate extreme heat impacts on crops. For instance, irrigated crop yields have been shown to be much more resistant to extreme heat than rainfed crops, indicating that yield sensitivity to heat depends on the availability of moisture^16,17^. However, wet conditions (from irrigation or rainfall) often cool heat extremes^18,19^ and raise atmospheric humidity^20,21^. This dependence between heat and moisture raises questions of whether crop impacts are caused by heat itself, or its connection to rainfall, soil moisture, and atmospheric aridity.

Understanding how heat impacts crops is essential to accurately project the risks of climate warming to global crop yields and evaluate the effectiveness of adaptation strategies. However, the extent of their impacts and the associated mechanisms remains uncertain. For example, studies disagree on the relative importance of temperature, soil moisture, and precipitation as predictors of crop yield variability^14,22,23^. In particular, the crop impacts of high temperature versus high-temperature and high humidity extremes, and their potentially differing connection to rainfall events, remains scarcely researched. This gap in understanding limits the ability to anticipate and prepare for yield impacts of projected increases in dry and humid heat extremes in various global breadbaskets.

**Figure 1:**
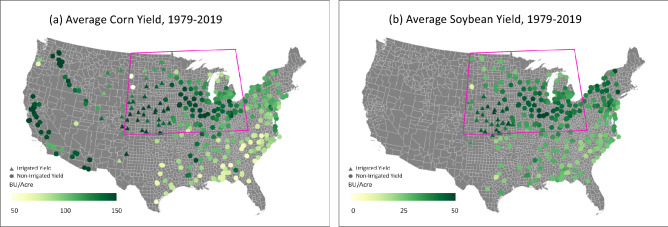
Average corn (**a**) and soybean (**b**) yields in bushels per acre from 1979 to 2019. The magenta outline indicates the commonly-defined U.S. Corn Belt. Circle dots are for non-irrigated yield, and triangles are for irrigated yield. Yield data are taken from the USDA National Agricultural Statistics Service (https://quickstats.nass.usda.gov) county level data. The maps are made with ArcGIS Pro 2.2.0 with the US base map downloaded from Census Bureau, https://catalog.data.gov/dataset/2019-cartographic-boundary-shapefile-current-census-tract-for-united-states-1-500000.

In this study, we compare the impacts of high-temperature but not high-humidity (extreme dry heat) versus high-temperature and high-humidity (extreme humid heat) on county-level corn and soybean yields in the United States. Our study covers the so-called U.S. Corn Belt (see Fig. 1, outlined regions), that produces about one third of the world’s corn and soybeans and is projected to become drier and warmer on a seasonal time scale^8^. We determine extreme dry and humid heat exposures, measured as the number of days exceeding certain dry and wet-bulb temperature thresholds (i.e., the 90th or 95th percentiles), and then use multiple regression to assess the relationship between these extreme heat exposures and crop yields. Wet-bulb temperature is a metric of compound high humidity and high temperature, a strong predictor of diminished agricultural labour productivity, negative human health impacts, and decreased productivity of other large mammals such as dairy cattle^2,3,5^. However, the impacts of humid heat extremes on crop yields as quantified using wet-bulb temperature are yet to be elucidated. Dry and wet-bulb temperatures, which are readily available or easy to calculate from existing observational data, are direct and highly consequential measures of extreme dry and humid heat conditions. We show that while dry heat extremes strongly decrease the yields of both crops (by about double the existing estimates), humid heat extremes generally have little impact, even though their associated dry-bulb temperatures exceed reported thresholds for yield losses. We further demonstrate that humid heat extremes, but not their dry counterparts, tend to be preceded by rainy conditions. This indicates that the ultimate impact of dry heat extremes on crops depends on the co-evolution of rainfall, humidity, soil moisture, and temperature in the genesis of extreme heat conditions, with implications for projecting and adapting to future climate risk to crops.

## Results

We define extreme heat exposures as days where stations are exposed to temperatures at or above the local 90th or 95th percentiles of daily maximum dry-bulb temperature (extreme heat) and daily maximum wet-bulb temperature (extreme humid heat) during the extended growing season (May through September, or MJJAS) based on a 30-year climatological base period, 1981–2010. We further define dry heat extremes as days exceeding the dry-bulb temperature threshold but not the wet-bulb threshold. Both the 90th and 95th percentile thresholds are used to determine the sensitivity of our results to differing levels of extreme heat. The analysis period in this study is from 1979 to 2019, though the extreme thresholds are defined based on the 30-year climatological period.

**Figure 2:**
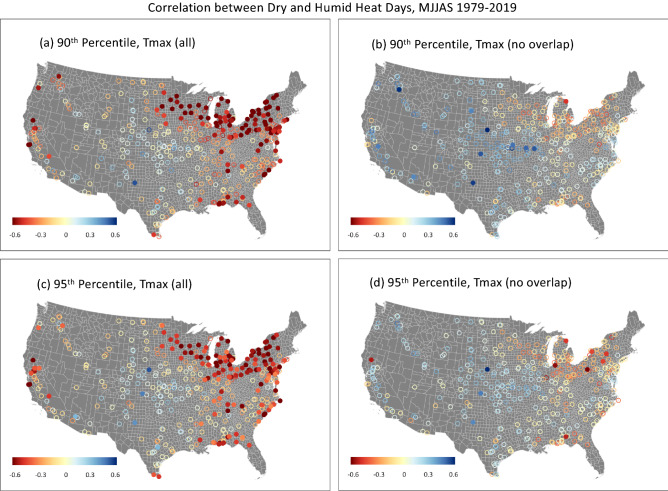
Correlation between dry and humid heat days for the corn growing regions. Year-to-year correlation coefficients for (**a**,**c**) between the number of extreme heat days (exceeding the 90th or 95th percentile threshold of maximum daily dry-bulb temperature, Tmax) and extreme humid heat days (exceeding the local 90th or 95th percentile thresholds of daily maximum daily wet-bult temperature, Twmax), and (**b**,**d**) between the number of extreme dry heat days (exceeding the 90th or 95th percentile threshold for Tmax, but not exceeding the corresponding threshold for Twmax) and extreme humid heat days. Correlation values significant at the 5% level using a two-sided Student-t test are shown as filled circles and the non-significant correlations are shown as open circles. The maps are made with ArcGIS Pro 2.2.0 with the US base map downloaded from Census Bureau, https://catalog.data.gov/dataset/2019-cartographic-boundary-shapefile-current-census-tract-for-united-states-1-500000.

To determine whether years with frequent high dry-bulb temperature (Tmax) days coincide with years with frequent high wet-bulb temperature (Twmax) days (high temperature and high humidity), we computed the correlation between the yearly MJJAS count of extreme Tmax days and extreme Twmax days at each station. We found significant positive year-to-year correlations over regions surrounding the Great Lakes, the east coast, and the Gulf coast, between extreme Tmax and extreme Twmax days (Fig. 2a,c). The positive correlations indicate the possibility that there are often co-occurrences of extreme Tmax and extreme Twmax days in these regions where moisture supply is high from the proximity to large water bodies. In order to differentiate the crop impacts of extreme dry and humid heat, we isolate the extreme dry heat days by removing the overlapping extreme Tmax days that also qualify for extreme Twmax days. Figure S1 shows the fraction of the extreme Tmax days that overlap with extreme Twmax days. In general, the overlapping days can be as high as 70% around the regions where the correlation between extreme heat and extreme humid heat days are high in Fig. 2a,c (e.g., around the Great Lakes). For the rest of the country, particularly the inland regions, the overlap fraction is usually less than 30%.

Once the days exceeding both the Tmax and Twmax thresholds are excluded from the set of extreme Tmax days, those remaining extreme Tmax days are considered extreme dry heat days, in contrast to the extreme Twmax days (extreme humid heat days). The year-to-year correlations between the total extreme dry heat days and humid heat days (Fig. 2b,d) are substantially reduced compared to Fig. 2a,c. The multicollinearity of humid and dry heat days as estimated using the variance inflation factor (VIF, see “Data and methods” and Fig. S2) is generally less than 2, indicating that the confounding influences of the dry and humid heat on crop yields are largely eliminated.

### Impacts of extreme dry and humid heat on crop yields

**Figure 3:**
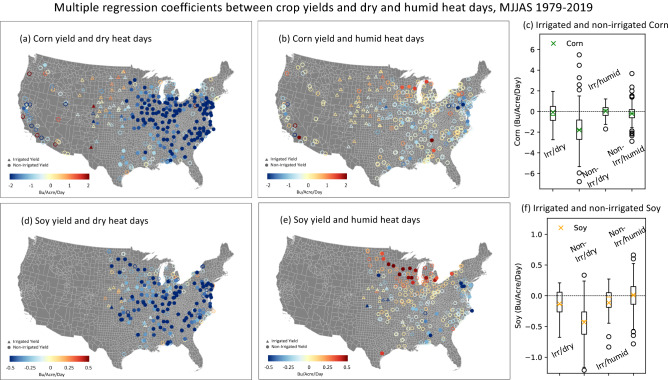
Multiple regression coefficients between crop yields and dry and humid heat days for all the counties with crop yield data. (**a**,**b**,**d**,**e**) Multiple regression coefficients between detrended corn (**a**,**b**) yields with detrended extreme dry (**a**) and humid (**b**) heat days (using the 95th percentile threshold) in bushels per acre per one heat day exposure for the period 1979–2019. (**d**) and (**e**) are the same as (**a**) and (**b**) but for detrended soybean yields. Significant values using a two-sided Student t-test at the 5% significance level are indicated in solid filled circles and triangles and non-significant values in open ones. Circles indicate non-irrigated yields and triangles indicate irrigated yield. (**c** and **f**) Box plots showing the interquartile range (boxes) and the data range (solid vertical lines, whiskers) of the regression coefficients for irrigated yields with dry heat (Irr/dry), non-irrigated yields with dry heat (Non-Irr/dry), irrigated yields with humid heat (Irr/humid) and non-irrigated yields with humid heat (Non-Irr/humid) for corn (**c**) and soy (**f**). The cross and horizontal line inside the box indicate the mean and median of the regression coefficients, respectively, and open circles in (**c**) and (**f**) indicate outliers. The maps are made with ArcGIS Pro 2.2.0 with the US base map downloaded from Census Bureau, https://catalog.data.gov/dataset/2019-cartographic-boundary-shapefile-current-census-tract-for-united-states-1-500000.

We next consider the impact of MJJAS seasonal extreme dry and humid heat exposures during the main agricultural season on corn and soybean yields. There are significant negative correlations between these crop yields and dry heat exposure, particularly in the eastern US (Figs. S3, S4a,c), indicating that dry heat extremes contribute to declines in corn and soybean yields. In contrast, the correlations between crop yield and humid heat exposure are small, generally not significant and even positive in certain locations (Figs. S3, S4b,d). To quantify the magnitude of the crop yield loss due to seasonal dry and humid heat exposures, we performed multiple regression of the detrended yield as a function of the number of both the dry and humid heat days at each station (see details in “Data and methods”). The multiple regression coefficients are shown in Fig. 3 for the 95th percentile threshold and in Fig. S5 for the 90th percentile threshold.

Exposure to extreme dry heat days significantly lowers corn yields by about -2 Bu/acre per day of dry heat exposure at the 95th percentile level for the MJJAS season. Critically, these yield sensitivities to dry heat exposure are about double those of past estimates that do not separate dry from humid heat extremes^10^. This finding suggests that the conflation of humid and dry heat extremes may lead to underestimated crop yield sensitivities to extreme dry heat. Furthermore, the negative impact is distinctly associated with dry heat exposure in regions without irrigation (Fig. 3a,d), as most of the irrigated yields (triangles) show non-significant regression coefficients with dry heat exposure, similar to that with humid heat exposure for both irrigated or non-irrigated yields (Fig. 3b,e). The box plots in Fig. 3c,f summarise the differing impacts of dry versus humid heat, as well as for irrigated and non-irrigated yields, indicating that the dry heat impact on yields are the most severe in non-irrigated regions while the dry heat impact on irrigated yields are similar to that of the humid heat. The reason for the similarity between irrigation and humid heat impact on yield will be discussed more below.

Since the average corn yields across all counties for this period range from 60 to 160 Bu/acre with a mean value of about 120 Bu/acre (see Fig. S7a, also see Fig. 1a), these yield reductions amount to about − 1.7% per day of dry heat exposure, or − 13% cumulatively for an average seasonal exposure of 7.5 days for the 95th percentile threshold. For soybeans, the regression coefficients with dry heat days is about − 0.5 Bu/acre per day of dry heat exposure at the 95th percentile level, which amounts to a reduction of approximately − 1.4% per day of dry heat exposure given the average yield of 35 Bu/acre for all counties and all years (see Figs. S3b and 1b), or − 10% cumulatively for an average seasonal exposure.

**Figure 4:**
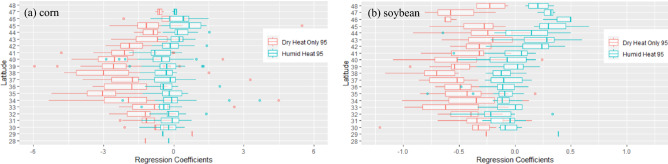
Latitudinal distribution of the multiple regression coefficients between yields and extreme dry/humid heat days. Shown are the box plots of the multiple regression coefficients at each latitude for corn (**a**) and soybean (**b**) yields. The red and blue boxes indicate the interquartile range of the regression coefficients at each latitude band for dry and humid heat, respectively, the vertical lines inside the boxes indicate the median values, and the horizontal lines (whiskers) indicate the ranges of the regressions. Dots are outliers.

To assess the latitude dependence of yield sensitivities to dry versus humid heat, we group the regression coefficients by latitude and assess their distributions (Fig. 4a,b). The negative regression coefficients between corn yield and dry heat exposure are particularly prominent in the latitudinal band between 32 and 45° North (Fig. 4a, red boxes), comprising the majority of the U.S. corn belt (Fig. 1). Corn yield sensitivities reach a median of − 3 Bu/acre (− 2.5% of average yield), with extreme sensitivities of up to − 5 to − 6 Bu/acre (− 4 to − 5%) per day of extreme dry heat exposure. The soy yield reduction shows similar sensitivity to latitudes, with extreme reductions exceeding − 1 Bu/acre/day (− 2.6%) at 38 and 40°N (Fig. 4b).

The lack of yield impacts from humid heat on corn and soybeans is consistent across the latitudinal domain. The median yield reductions due to humid heat exposure are close to zero across most latitudes (Fig. 4a,b cyan boxes) and clearly separate from the more negative interquartile range of yield sensitivities to dry heat. There are some beneficial effects of the humid heat for soy yield north of 42°N, with a median yield increase reaching 0.5 Bu/Acre per day of extreme humid heat exposure at 46°N. This beneficial effect of humid heat exposure can also be seen in Fig. 3e for Soybeans near the Great Lakes, likely a result of the yield benefits from a combination of heat accumulation in cooler climates without simultaneously high moisture demand.

The high negative yield sensitivities of both crops to dry heat relative to humid heat is generally highest between 34 and 41°N latitude, and the differences in sensitivities narrow toward both lower and (especially for corn) higher latitudes. For corn, this narrowing is driven by less negative yield sensitivities to dry heat exposure (Fig. 4a). Decreasingly negative yield sensitivities to dry heat at lower latitudes in the southern part of the domain have been previously attributed to farmer adaptation to higher climatological extreme heat^24^. The mirrored trend in the northern part of the domain could be due to yield benefits from greater heat accumulation in cooler northern climates.

Our results on the disparate impact of dry and humid heat on corn and soybean yields are not sensitive to the choice of extreme heat threshold (compare the 95th percentile threshold in Fig. 3 to Fig. S5 for the 90th percentile threshold) or the seasonal timing of the extreme heat exposures (Fig. S6). Figure S6 demonstrates that corn yields are slightly more sensitive to dry heat extremes during the latter part of the season (JAS) compared to the earlier part (MJ). However, in both the early and late season, there is significant yield loss due to dry heat but not due to humid heat. We also applied a national scale fixed-effect space–time linear model on the non-detrended dataset, which allows inter-county differences and long-term time trends to be included in the regression model, as detailed in the “Data and methods” section, for robust check against the multiple regression applied to a fixed location on detrended data set. The results are consistent with the findings in Figs. 3 and 4 in terms of the differing magnitude of yield loss due to extreme dry and humid heat exposures (Table 1).

**Table 1:** Comparison of multiple regression between yield and heat days using two different methods.

Multiple regression coefficients between yield and heat days		Dry heat days coefficients (beta1)	Humid heat days coefficients (beta2)
Multiple regressions (area average)	Corn yield	− 1.80 Bu/Acre/day	− 0.20 Bu/Acre/day
	Soybean Yield	− 0.43 Bu/Acre/Day	0.01 Bu/Acre/day
Fixed effects linear model	Corn Yield	− 1.58 Bu/Acre/day	− 0.01 Bu/Acre/day
	Soybean yield	− 0.52 Bu/Acre/day	0.001 Bu/Acre/day

The first uses the area-averaged multiple regression across all non-irrigated stations using detrended yield data at each county and dry and humid heat days for all stations within that county (Fig. 3) and the second uses the space–time fixed effects linear model applied to non-detrended data for all non-irrigated stations (Eq. 3).

### Deciphering the distinct impacts of dry and humid heat on crop yields

While the negative impacts of dry heat on crop yield are generally consistent, albeit stronger, with previous studies showing the adverse effect of high temperature (greater than 30 °C) on crop yields^10^, previous studies have not directly assessed the lack of negative correlation with extreme humid heat exposure. To further explore the underlying reasons for the distinct difference between extreme dry and humid heat impacts on crop yields we perform the following additional analyses.

**Figure 5:**
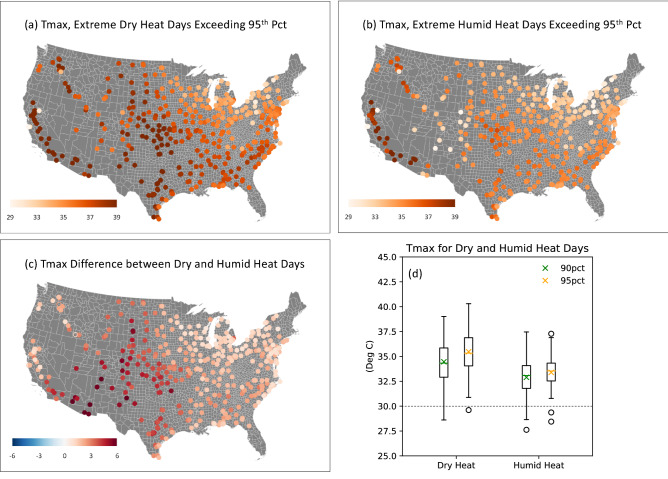
Average Tmax for extreme Dry and Humid Heat Days for the U.S. corn growing regions. (**a**) Average Tmax for all extreme dry heat days exceeding the local 95th percentile of Tmax, but not exceeding the local 95th percentile of Twmax, (**b**) average Tmax for all extreme humid heat days exceeding the local 95th percentile of Twmax, and (**c**) differences between average Tmax for dry and humid heat days (**a**,**b**), for the period 1979–2019. (**d**) Box plots for the distribution of mean Tmax above the 90th (green) and 95th (orange) percentiles at stations with significant corn yield losses due to dry heat (significant negative regression coefficients in Figs. 3a and S5a). Boxes and whiskers show the interquartile range and data range, respectively, of average Tmax for dry (left) and humid (right) heat days. The cross markers and horizontal lines inside the boxes represent the mean and median, respectively, and open circles are outliers. The maps are made with ArcGIS Pro 2.2.0 with the US base map downloaded from Census Bureau, https://catalog.data.gov/dataset/2019-cartographic-boundary-shapefile-current-census-tract-for-united-states-1-500000.

First, we ask whether the differing impacts occur because extreme humid heat days are associated with lower daily maximum dry-bulb temperatures compared to the extreme dry heat days. We calculate the average Tmax across all dry heat days and that for all humid heat days, as well as their differences (Fig. 5a–c). The average Tmax associated with extreme humid heat (Fig. 5b) are cooler than those for extreme dry heat (Fig. 5a). When we consider only stations with a significant corn yield reduction due to dry heat (Fig. 3a), we find a median Tmax over the 95th percentile of about 33 °C for humid heat and 35 °C for dry heat (Fig. 5d, orange line inside the box). Overall, as shown in Fig. 5c, the differences in Tmax for dry versus humid extremes are generally less than 3 °C, except in the climatologically drier central plains where irrigation is more prevalent (see the triangles in Fig. 1). For the region with the most significant yield reduction due to dry heat (Fig. 3a,d), the dry-bulb temperature differences range between 0 and 2 °C (Fig. 5c).

Further, Fig. 5d shows that both dry and humid heat extremes have average daily Tmax well above the ~ 30 °C empirical threshold for negative crop yield impacts^10^, yet we find no widespread yield reductions due to humid heat exposure. Additionally, the Tmax range for dry heat exceeding the 90th percentile (Fig. 5d, green markers for dry heat) overlaps substantially with that for humid heat exceeding the 95th percentile (Fig. 5d, orange markers for humid heat), yet dry heat above the 90th percentile leads to significant yield losses (Fig. S5), whereas humid heat above the 95th percentile does not. In other words, while humid heat extremes are somewhat less ‘hot’ than dry heat extremes, they are hot enough that literature to date suggests they should cause significant yield reductions, and nevertheless they do not. This finding suggests that temperature differences alone do not explain the dramatic differences in the yield reduction seen in Fig. 3.

While our results are consistent with the idea that moisture demand (vapor pressure deficit) is a key driver of crop yields, an alternate explanation for the low yield sensitivity to extreme humid heat could relate to moisture supply, rather than demand. Previous studies have shown that limiting soil moisture supply during high temperatures may be the ultimate reason for the adverse effect of heat stress on crops^23,25^. We next address the question of whether the disparate impacts of dry and humid heat on crop yields are related to differences in moisture availability by examining average precipitation for the 3 days immediately preceding and following extreme Tmax or Twmax events.

**Figure 6:**
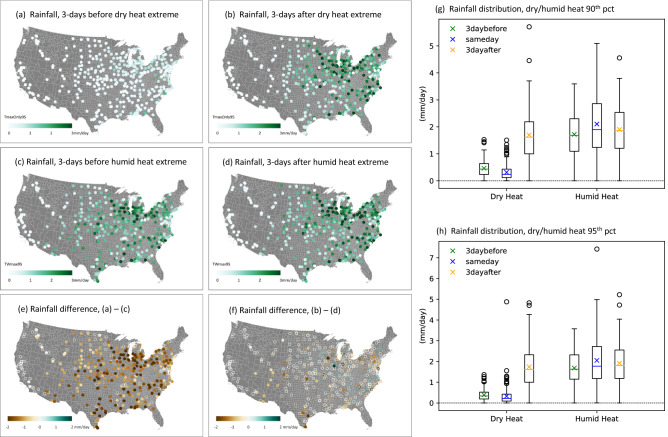
Rainfall before and after the extreme heat events. Rainfall averaged over 3 days before (**a**) extreme dry heat days and (**c**) extreme humid heat days, and (**e**) their differences. (**b**), (**d**) and (**f**) are the same as (**a**), (**c**) and (**e**) except for rainfall averaged over the 3 days following the extreme dry and humid heat days. Solid (open) circles in (**e**) and (**f**) indicate significant (non-significant) differences at the 95% confidence level using two-sided Student t-test. Rainfall units are in mm/day. (**g**) and (**h**) Box plots showing precipitation distribution for all stations with significant negative regression coefficients in Fig. S5a for the 90th percentile extreme heat threshold (**g**), and in Fig. 3a for the 95th percentile threshold (**h**), for 3-days before (green marks), same day (blue), and 3-days after (orange) the extreme dry and humid heat event. The maps are made with ArcGIS Pro 2.2.0 with the US base map downloaded from Census Bureau, https://catalog.data.gov/dataset/2019-cartographic-boundary-shapefile-current-census-tract-for-united-states-1-500000.

We find substantial differences in accumulated precipitation over the three days prior to extreme dry and humid heat exceedances, with more than double the amount of daily precipitation prior to humid heat days (Fig. 6c) compared to dry heat days (Fig. 6a) across the vast majority of the crop growing regions, except in the western US. This difference (Fig. 6e) uniquely applies to the lead-up to extreme heat events; average precipitation for the three days immediately following the extreme dry and humid heat exceedances (Fig. 6b,d) are not consistently different from each other (Fig. 6f). This result is somewhat surprising, as one would expect extreme wet-bulb temperature, with its associated high atmospheric humidity, could lead to more precipitation. This sequence of higher rainfall before a humid heat event suggests that the abundance of moisture prior to the heat event may determine whether it is humid or dry. Figure 6g,h show the rainfall distribution for 3-days before and after the extreme heat, as well as on the day of the extreme heat, for all stations that show significant yield reductions in Figs. S5a and 3a. There is a clear separation of rainfall across the stations for three days before and on the same day of the dry heat as compared to that for three days after the dry heat. However, there is no difference for precipitation associated with 3-days before and after, or during the extreme humid heat, highlighting the distinctive role of the lack of rainfall linking to yield loss during extreme dry heat.

Extreme dry heat is generally preceded by low daily precipitation in the days leading up to the dry heat (Fig. 6a), and thus relatively dry surface conditions and low atmospheric humidity. On the other hand, extreme humid heat days are generally preceded by higher daily precipitation (Fig. 6c), and thus wetter surface conditions and higher atmospheric humidity. This finding indicates that the moist thermodynamic characteristics of extreme heat events determine the magnitude and even the direction of their crop impacts. Specifically, our results suggest that crop impacts linked to extreme heat are not induced by the extreme heat itself, but by a multivariate extreme involving high dry-bulb temperatures preceded by low rainfall and accompanied by low atmospheric humidity, and likely low soil moisture.

By contrasting the crop yield sensitivities for dry and humid heat extremes, our study helps clarify the causality of heat impacts on crop yields. The absence of yield losses from exposure to humid heat days with Tmax > 30 °C suggests that extreme heat mainly reduces yields through indirect moisture stresses. This conclusion is consistent with the fact that damaging effects of dry heat extremes in this study (and in others investigating yield impacts of temperature irrespective of humidity) occur at lower temperatures (~ 30 °C) than experimental thresholds for direct thermal impacts^12,13^. However, we note that dry and humid heat impacts may further differ through a crop-climate feedback whereby stomatal closure during dry heat amplifies local canopy heating, whereas sustained transpiration during humid heat can locally buffer regional high temperatures^15,26,27^. This feedback may boost local crop canopy temperatures during dry heat compared to air temperatures measured at weather stations^28^, which may be limited during humid heat.

The close relationship between higher rainfall directly preceding heat events and reduced crop yield loss further suggests that the timing of the rainfall relative to high heat exposure is crucial to impacts on crop yields, a novel finding of our study. Previous studies suggest that moisture supply shortages associated with dry soil conditions coupled with high temperature exposure are the key ingredients for crop yield reduction, rather than extreme heat exposure alone^23,25,29–31^. Our results extend these past findings by showing how the specific combination of rainfall, humidity, and heat determines whether extreme temperatures lead to benign or damaging impacts on crops. This may help explain the previously-reported weak correlation between crop yields and seasonal mean precipitation, a metric that is agnostic of the timing of rainfall and humidity relative to extreme heat^10,13^. However, whether the lack of yield losses during humid heat extremes is attributable to plant-available soil moisture from the antecedent rainfall, as opposed to the ensuing atmospheric humidity, merits further research.

The lack of strong negative impact of extreme humid heat on crop yields and the beneficial effect of rainfall immediately prior to high heat exposure begs the question of how irrigation may play a role in the relationship between dry and humid heat impact on crop yields. In Fig. 3, the yield regression coefficients due to extreme dry and humid heat exposures and the irrigated corn and soybean yields are shown as triangles for the irrigated regions, which covers mostly the Dakotas and Nebraska. For both corn and soybeans, the irrigated regions show non-significant yield loss associated with both dry and humid heat exposures (Fig. 3c,f), with the average regression coefficients close to zero for corn and slightly negative for soy, consistent with previous studies indicating that irrigation reduces the negative impacts of the high heat stress^16,29,32^. But our results here raise the possibility that irrigation effectively converts regional dry heat to local humid heat in the irrigated crop canopy, as irrigation water is readily evaporated and transpired under hot conditions, humidifying the boundary layer.

Importantly, the climatology of rainfall-heat sequences will likely be altered by changing mean seasonal aridity^8^, rainfall intensification^33,34^, and land–atmosphere coupling in a warming climate^21,31^, with complex implications for crop production. For example, positive trends in extreme dry heat incidence are already detectable for the U.S. corn growing region (Fig. S7). We find a significant upward trend in the frequency of extreme dry heat days, increasing about 3 days for the 90th percentile threshold and 1.5 days for the 95th percentile threshold over the past 41 years. There is no significant increase in the frequency of extreme humid heat days over the region using either the 90th or 95th percentile threshold during this period. A key implication of our study is that the widespread expectation of yield increases from warming in cool climates may not come to be if hot days become drier^35^. On the other hand, projected future increases in humid heat frequency^4^ could limit crop impacts of warming. Further, possible changes in weather sequences associated with large-scale dynamics are an additional wildcard. Thus, potential future changes in compound rainfall-heat sequences and their crop impacts remain uncertain in both their frequencies and severity.

## Discussion

This study explores the distinct impacts of extreme dry and humid heat exposure on crop yields in US corn growing regions. While our results support previous findings on the adverse effect of extreme high temperature exposure on crop yields, we also find that complex interactions of precipitation, soil moisture, temperature, humidity, and vegetation act to reduce heat impacts for humid heat compared to dry heat. This is the first study to illustrate the different dry versus humid heat impact on crop yields using simple and common meteorological measures of dry and wet-bulb temperatures. Our results further indicate that the ultimate crop impacts of heat depend on the sub-seasonal evolution of heat events, in that humid heat tends to be immediately preceded by more rainfall.

Future research may further attribute the greatly-reduced yield loss from humid heat extremes to atmospheric humidity or soil moisture and vegetation supply, and their interactions. Other metrics (e.g. combinations of not just frequency but also intensity) and variables (e.g. vapour pressure deficit and shortwave radiation) will also be instructive. While we find that more precipitation tend to lead the humid heat extreme but not dry heat extremes, future studies could explore more fully the two-way interactions between leading precipitation and dry and humid heat via atmospheric dynamics (associated for example with moisture advection), and more local moisture recycling via the land surface (soil moisture). Since these interactions may have characteristic timescales longer than the 3 day lead and lag periods explored here, more research is needed on how yields are impacted by multivariate interactions between subseasonal precipitation and temperature at these longer timescales. Furthermore, the yield effects of relative proximity to large sources of moisture climatologically and during weather sequences surrounding heat extremes, from the Gulf of Mexico and Great Lakes especially, is worthy of further research. More research is needed on heat impacts at different stages in plant phenological cycles, and on other crops and regions. In addition, greater attention is needed to project how these relationships may change in the future and understand the likely crop consequences. Understanding the underlying mechanisms of the difference between the dry and humid heat impacts on crop yields is essential to adapt crop genetics and management to changing joint climatology of heat and moisture.

Our findings point to the need of more crop-centric future research. For example, further research is needed on whether the seemingly small difference in maximum dry bulb temperatures between dry and humid heat events is actually important for crops, given the nonlinearities of crop response and Clausius-Clapeyron at high dry bulb temperatures. Indeed, the fact that maximum dry bulb temperature is only 1–2 °C higher on dry heat than humid heat days over the core of the corn growing region begs the question of whether part of the reason the temperature difference is so small is because the corn/soybean plants are transpiring a lot of water on these dry heat days to the environment, thus capping the dry-bulb temperature^27^. Perhaps they sacrifice potentially-precious water—beyond what photosynthetic need and associated stomatal opening transpiration dictates—precisely to thermoregulate and avoid dangerous impacts from the higher dry bulb temperature that would otherwise exist, especially at the canopy level. These transpiration-related questions are especially interesting given the larger dry bulb temperature differences on dry heat and humid heat days in areas where crop production is less intensive. Along similar lines, it would be interesting to ask whether during rare years when the corn-belt crops truly failed catastrophically, such as during persistent dry years of the Dust Bowl period, dry bulb maximum temperatures spiked a lot higher than what we report here. Of course, other related factors besides reduced crop production and transpiration could be responsible for the larger temperature differences further west (Fig. 5c); adiabatic descent from mountains might elevate dry bulb temperature a lot higher on dry days than on humid days, soil and forest moisture might be too low to depress dry bulb temperature and climatological advection of specific humidity might be too low. But the role of crop transpiration in our findings is worthy of further research, and studies that regionally disaggregate the results presented here might prove informative.

Our results suggest that one way to alleviate the negative dry heat impact on corn and soybean yields is through irrigation^36^, preferably immediately before the extreme heat occurs, emphasizing the importance of sub-seasonal prediction of dry heat extremes. However, under increased future temperatures, irrigation will not only increase the costs and labour requirements for farming in some regions, but will be limited by water availability due to future drying and unsustainable withdrawals from certain aquifers^37^. Thus, other adaptive strategies will need to be considered including changing planting times or using fast-maturing varieties to avoid peak summer dry heat, altering sowing densities and thus crop canopy thermodynamics and crop water-use efficiency, and developing new jointly drought and heat resistant cultivars. Most fundamentally, our study illustrates how future research into climate-adaptive cropping should consider nuanced changes in the climatology of rainfall, humidity, and heat as climate change proceeds.

Finally, while we find that crop yields are largely resistant to humid heat, increased humid heat exposure will pose significant health risks to outdoor agricultural workers^38,39^. Therefore, adaptation and research attention should be paid to future increases in humid heat exposure for agricultural workers and the related impact on agricultural production as the atmosphere warms.

## Data and methods

### Temperature and yield data

We use the UK Met Office Hadley Centre global sub-daily station observations (HadISD) version 3.1.1.202004p^40,41^ to obtain the daily maximum dry-bulb temperature (Tmax) and daily maximum wet-bulb temperature (Twmax) from 1979 to 2019 in this study. We use hourly HadISD dry-bulb temperature, and calculate the wet-bulb temperature using specific humidity, elevation, and mean sea level pressure, as in ref.^3^. We use the Davies-Jones method^42^ to calculate hourly wet-bulb temperatures, and apply the ref.^43^ implementation of this method using the Matlab code in ref.^44^. This method minimizes error at high temperatures^2,4,45^. Daily maximums are calculated as the maximum of hourly dry- and wet-bulb temperatures.

The extreme heat days for the crop growing season, from May to September, are defined as those days when Tmax exceeds the local 90th or 95th percentile threshold of Tmax, but Twmax does not exceed the same threshold for Twmax (dry heat days) and Twmax exceed the local 90th and 95th percentile threshold (humid heat days) values based on the 30-year base period, 1981–2010. Figure S8 shows the 90th and 95th percentile local threshold values for Tmax and Twmax based on the 30-year base period, 1981–2010. The higher the number of days exceeding the threshold values, the more extreme heat exposure for the crops. We use percentile thresholds here instead of absolute temperature thresholds (e.g. 30 °C), because crops growing in a region may have adapted to the local climate to some degree^45^. Also, while dry-bulb temperature thresholds have been empirically established^10^, no such basis yet exists for wet-bulb temperatures. We apply quality control to the station data in the same way as in ref.^3^, in that we require data for at least 90% of the days in 90% of the months between 1979 and 2019. Stations that do not meet this threshold are removed from the analysis. The analysis here only uses the stations that are located within the county boundary that provides crop yield data (see Fig. 3 for the stations used for corn and soybeans). To assess the relationship between yield and dry and humid heat days, we compute the correlation or construct the multiple regression between yield in a given county and the extreme heat days for all stations within that county.

The U.S. corn and soybean yield data are taken from USDA National Agricultural Statistics Service (https://quickstats.nass.usda.gov) county level data from 1979 to 2019. Both non-irrigated and irrigated yields are used for corn and soybeans, as indicated by the circles and triangles in Fig. 1, respectively. Given the large trend in yield over the years (Fig. S7a,b) due to non-climate related factors, we first linearly detrend the corn and soybean yields by first calculating the linear regression of the yield data to time (years) and then subtract the fitted line from the yield data. Dry and humid heat day time series are likewise linearly detrended to isolate the influence of interannual variability from long term trends (Fig. S7c–f).

### Analysis methods

Pearson’s correlations are used to calculate the correlation between detrended yields and extreme heat days as:

1 $$r\left(yield,heatdays\right)=\frac{{\sum }\left(y{ield}^{*}\times {heatdays}^{*}\right)}{\sqrt{{\sum }{\left({yield}^{*}\right)}^{2}{\sum }{\left({heatdays}^{*}\right)}^{2}}}$$

where yield* is the detrended yield anomalies with respect to the long term averages and heatdays* is the detrended extreme dry or humid heat day frequency anomalies with respect to their long term averages. Pearson correlations between detrended dry heat days and corn and soybean yields are significantly negative across most of the corn and soybean growing regions in the U.S. at both the 90th (Fig. S3a,c) and 95th (Fig. S4a,c) percentile thresholds. On the other hand, the same yield correlation with extreme humid heat days are much weaker and mostly non-significant, and even positive in some regions (Figs. S3b,d and S4b,d).

We further apply multiple regression models linking yield to dry and humid heat exposure for each county of the form:

2 $${\widehat{yield}}_{t}= {\beta }_{0}+{\beta }_{1}{DHD}_{t}+{\beta }_{2}{HHD}_{t}$$

in which *DHD* denotes dry heat days, *HHD* denotes humid heat days, ${\beta }_{1}$ denotes the yield sensitivity to dry heat days, ${\beta }_{2}$ denotes yield sensitivity to humid heat days, and subscript *t* denotes years. We fit these models separately for corn and soy. The significance of the regression coefficient *β*_*1*_ and *β*_*2*_ is determined by dividing the estimated coefficient over the standard errors of this estimate and then using the two-sided t-test at the 95th confidence level.

As a robustness check, we also apply a national scale fixed-effect linear model on the non-detrended dataset of the form:

3 $${\widehat{yield}}_{i,t}={C}_{i}+{T}_{t}+{\beta }_{1}{DHD}_{i,t}+{\beta }_{2}{HHD}_{i,t}$$

in which ${C}_{i}$ and ${T}_{t}$ are fixed effects for baseline inter-county differences and long-term time trends, respectively, and subscripts *i* and *t* denote counties and years. National-scale yield sensitivities to dry and humid heat estimated using this method are consistent with the national average of county-scale sensitivities estimated using multiple regression.

To measure the multicollinearity between the two independent variables in Eq. (2,3), the dry heat days (DHD) and humid heat days (HHD), we computed the variance inflation factor (VIF) as follows:

4 $$VIF=\frac{1}{1-{R}^{2}}$$

where R is the correlation coefficient between dry heat days and humid heat days.

## Supplementary Information

Supplementary Figures.

## Data Availability

The UK Met Office Hadley Centre HadISD sub-daily station dataset is publicly available at https://www.metoffice.gov.uk/hadobs/hadisd/. The MatLab code used to calculate the hourly wet-bulb temperature using specific humidity, elevation, and mean sea level pressure, is available at https://github.com/bobkopp/WetBulb.m. The U.S. corn and soybean yield data used in this study are publicly available from US Department of Agriculture’s National Agricultural Statistics Service website: https://quickstats.nass.usda.gov.
