# Supplementary material for: Contrasting impacts of dry versus humid heat on US corn and soybean yields
Source: Sci Rep. 2023 Jan 13;13:710. doi: 10.1038/s41598-023-27931-7 (PMC9839719; doi:10.1038/s41598-023-27931-7)
Supplement: Supplementary file 1 — Supplementary Figures. [file 41598_2023_27931_MOESM1_ESM.docx]

**Supplementary Information (SI) for**

**Contrasting Impacts of Dry Versus Humid Heat on US Corn and Soybean Yields**

Mingfang Ting^1*^, Corey Lesk^1-3^, Chunyu Liu^1,4^, Cuihua Li^1^, Radley M. Horton^1^, Ethan D. Coffel^5^, Cassandra D.W. Rogers^6^, and Deepti Singh^6^

^1^ Lamont-Doherty Earth Observatory, Columbia University, Palisades, New York

^2^ Neukom Institute for Computational Science, Dartmouth College, Hanover, NH, USA.

^3^ Department of Geography, Dartmouth College, Hanover, NH, USA

^4^School of Oceanography, Shanghai Jiao Tong University, Shanghai, China

^5^ Department of Geography and the Environment, Syracuse University, New York, New York

^6^ School of the Environment, Washington State University, Vancouver, Washington

*Mingfang Ting

**Email:**  [ting@ldeo.columbia.edu](mailto:ting@ldeo.columbia.edu)

**SI Figures S1 to S****8**

**Figure** **S1.** The fraction of dry heat days that are also humid heat days for those days exceeding the 90^th^ (a) and 95^th^ (b) percentile thresholds of the daily maximum temperature, Tmax, for dry heat days, and the daily maximum wet bulb temperature, TWmax, for humid heat days over the U.S. corn growing regions for MJJAS, 1979 - 2019. The maps are made with ArcGIS Pro 2.2.0 with the US base map downloaded from Census Bureau, https://catalog.data.gov/dataset/2019-cartographic-boundary-shapefile-current-census-tract-for-united-states-1-500000.

**Figure S2.** The variance inflation factor (VIF, see Eq. 4 in Methods) between the two independent variables, dry heat days and humid heat days, in the multiple regression between yield and extreme heat days (see Eq. 3 in methods). Left panels show the VIF between dry and humid heat days (with overlapping days) that exceeds the 90^th^ (a) and 95^th^ (c) daily Tmax and Twmax percentile thresholds. Right panels show the same but with overlapping Tmax and Twmax days removed from the set of Tmax days. As expected, the VIF reduces when the overlapping days are removed. But even with the overlapping days included, the VIF values are generally less than 3. The maps are made with ArcGIS Pro 2.2.0 with the US base map downloaded from Census Bureau, https://catalog.data.gov/dataset/2019-cartographic-boundary-shapefile-current-census-tract-for-united-states-1-500000.

Figure S3. Correlation coefficients between detrended extreme dry (a,c) and humid (b,d) heat days with detrended corn (a,b) and soybean (c,d) yields for 1979-2019. Extreme dry heat days are defined as days exceeding the local 90^th^ percentile threshold for Tmax but not exceeding the same threshold for Twmax, and extreme humid heat days are defined as days exceeding the local 90^th^ percentile threshold of Twmax. The station data points shown represent the HadISD observation stations that are located within the county that provided the yield data. Solid fill circles (non-irrigated yields) and triangles (irrigated yields) indicate correlations exceeding the 95% confidence level using a two-tailed Student t-test. Non-significant correlations are indicated by open circles (non-irrigated yield) and open triangles (irrigated yield). The maps are made with ArcGIS Pro 2.2.0 with the US base map downloaded from Census Bureau, https://catalog.data.gov/dataset/2019-cartographic-boundary-shapefile-current-census-tract-for-united-states-1-500000.

**Figure S4.** Correlation coefficients between detrended extreme dry (a,c) and humid (b,d) heat days with detrended corn (a,b) and soybean (c,d) yields for 1979-2019. Extreme dry heat days are defined as days exceeding the local 95^th^ percentile threshold for Tmax but not exceeding the same threshold for Twmax, and extreme humid heat days are defined as days exceeding the local 95^th^ percentile threshold of Twmax. The station data points shown represent the HadISD observation stations that are located within the county that provided the yield data. Solid fill circles (non-irrigated yields) and triangles (irrigated yields) indicate correlations exceeding the 95% confidence level using a two-tailed Student t-test. Non-significant correlations are indicated by open circles (non-irrigated yield) and open triangles (irrigated yield). The maps are made with ArcGIS Pro 2.2.0 with the US base map downloaded from Census Bureau, https://catalog.data.gov/dataset/2019-cartographic-boundary-shapefile-current-census-tract-for-united-states-1-500000.

**Figure S5**. (a-b, d-e) Multiple regression coefficients between detrended corn (a,b) yields with detrended extreme dry (a) and humid (b) heat days (using the 90^th^ percentile threshold) in bushels per acre per one heat day exposure for the period 1979-2019. (d) and (e) are the same as (a) and (b) but for detrended soybean yields. Significant values using a two-sided Student t-test at the 95% confidence level are indicated in solid filled circles and triangles and non-significant values in open ones. Circles indicate non-irrigated yields and triangles indicate irrigated yield. (c and f) Box plots showing the interquartile range (boxes) and the data range (solid vertical lines, whiskers) of the regression coefficients for irrigated yields with dry heat (Irr/dry), non-irrigated yields with dry heat (Non-Irr/dry), irrigated yields with humid heat (Irr/humid) and Non-irrigated yields with humid heat (Non-Irr/humid) for corn (c) and soy (f). The cross and horizontal line inside the box indicate the mean and median of the regression coefficients, respectively, and open circles in (c) and (f) indicate outliers. The maps are made with ArcGIS Pro 2.2.0 with the US base map downloaded from Census Bureau, https://catalog.data.gov/dataset/2019-cartographic-boundary-shapefile-current-census-tract-for-united-states-1-500000.

**Figure S6.** Multiple regression coefficients between corn yield and extreme dry (a,c) and humid (b,d) heat days exceeding the 95th percentile thresholds for early season (MJ, a,b) and the late season (JAS, c,d). The regression coefficients that exceed the 95% confidence level using a two-tailed Student t-test are shown as solid fill. Circles are for rainfed and triangles for irrigated yields. The maps are made with ArcGIS Pro 2.2.0 with the US base map downloaded from Census Bureau, https://catalog.data.gov/dataset/2019-cartographic-boundary-shapefile-current-census-tract-for-united-states-1-500000.

**Figure S7.** Averaged crop yield and heat day frequency as a function of time from 1979-2019. (a) and (b) show the corn and soybean yields averaged across the entire US corn and soy growing regions, (c) to (f) show thee area averaged extreme dry (c,e) and humid (d,f) heat days for MJJAS that exceeded the 90^th^ (c,d) and 95^th^ (e,f) percentiles. Blue lines indicate the linear trend regression line and shaded regions indicate the 95% confidence intervals of the regression lines.

**Figure S8.** The 90^th^ (a, b) and 95^th^ (c,d) local threshold values for Tmax (a,c) and Twmax (b,d) based on the 30-year base period from 1981-2010. The maps are made with ArcGIS Pro 2.2.0 with the US base map downloaded from Census Bureau, https://catalog.data.gov/dataset/2019-cartographic-boundary-shapefile-current-census-tract-for-united-states-1-500000.
